# The Role of Religion, Spirituality and/or Belief in Positive Ageing for Older Adults

**DOI:** 10.3390/geriatrics3020028

**Published:** 2018-06-08

**Authors:** Joanna Malone, Anna Dadswell

**Affiliations:** 1 School of European Culture and Languages, University of Kent, Canterbury CT2 7NZ, UK; 2 Faculty of Health, Social Care and Education, Anglia Ruskin University, Bishop Hall Lane, Chelmsford CM1 1SQ, UK; anna.dadswell@anglia.ac.uk

**Keywords:** positive ageing, religion, spirituality, belief, health, well-being

## Abstract

(1) Background: The concept of positive ageing is gaining recognition as an approach to better understand the lives of older adults throughout the world. Positive ageing encompasses the various ways in which older adults approach life challenges associated with ageing and how certain approaches allow older adults to age in a more positive way. This paper makes a contribution to the field by examining the role of religion, spirituality and/or belief in relation to positive ageing; (2) Methods: Qualitative focus groups with 14 older adults living in West London explored the role and importance religion, spirituality and/or belief held in their everyday lives and how this could be incorporated into the idea of positive ageing; (3) Results: Religion, spirituality and/or belief were found to play a number of roles in the everyday lives of the older adults, including being a source of strength, comfort and hope in difficult times and bringing about a sense of community and belonging; (4) Conclusion: This paper argues that religion, spirituality and/or belief should be included within positive ageing literature and be viewed as a type of support (amongst multiple others) that helps older adults to live positive lives despite the many challenges of ageing.

## 1. Introduction

The concept of positive ageing is gaining attention in the literature as an approach to health and well-being that incorporates a range of factors in the lives of older adults. These factors encourage an understanding of older adults as rounded individuals—rather than a series of health issues—and go beyond the aspiration to promote only physical and mental health, which is sometimes unachievable in older age. One factor that has received little attention in the literature on positive ageing is the role of religion, spirituality and/or belief. Religion, spirituality and/or belief are still centrally important for many people, providing structure, meaning and understanding to everyday life, as well as support through life challenges [1,2]. The literature indicates the potential benefits of religion, spirituality and/or belief for health, well-being and quality of life [3], particularly for older adults [4,5]. However, Wilkinson and Coleman state that “… many people in the UK today have vague, individualised spiritual beliefs that are unattached to religious doctrines and that may be a relatively poor and untested resource for coping with ageing” [6] (p. 340). This highlights the need for further investigation into the role that religion, spirituality and/or belief may have in promoting positive ageing for older adults in the UK. As such, this article brings together the relevant literature and considers its application in relation to a qualitative exploratory study with older adults in London in order to make recommendations for future research and practice. Given the location of the study, the paper focuses predominantly on literature from Western cultures and societies, though the authors acknowledge the diversity of religion, spirituality and/or belief around the world and the interesting ways in which this might influence differences in positive ageing.

This introduction will first discuss some conceptual understandings of the issues at stake, before addressing the context of complexity and change followed by the influence of religion, spirituality and/or belief on health, well-being and positive ageing. The article will then turn to the methodology, followed by the findings, discussion and conclusion.

### 1.1. Conceptual Understandings of Positive Ageing, Religion, Spirituality and Belief

As noted, the concept of positive ageing is one which has been growing in academic and public debate in recent years. A number of studies have been conducted and a number of papers written which explore positive ageing. The meaning of positive ageing is not clear cut, but a definition from the Centre for Positive Ageing states:

*‘Positive Ageing’ denotes the aspirations of individuals and communities to plan for, approach and live life’s changes and challenges as they age and approach the end of their lives, in a productive, active and fulfilling manner. The focus embraces the idea of making the most of opportunities, innovations and research which promote a person’s sense of independence, dignity, well-being, good health and enable their participation in society*.

[7] (p. 5)

From this definition, the idea of positive ageing can encompass a wide variety of different aspects in everyday life which can facilitate or inhibit positive ageing. For example, Chong et al. explored the thoughts and lay theories of middle-aged and older adults in Hong Kong. For them, positive ageing meant maintaining relationships, having good family and social support, and active participation in a number of activities and within the community. Having a positive attitude towards themselves and having a sense of purpose in life were also deemed important. Furthermore, the older adults also discussed the role of religion and stressed that “the most important tenets of religion were the moral codes, not the matters of faith, and they believed that all religious belief provided a person with positive attitudes towards life” [8] (p. 259).

Similar notions to positive ageing have been used in academic discourse, the most prominent of which is ‘successful ageing’. Rowe and Kahn’s work on successful ageing includes three main aspects: “low probability of disease and disease-related disability, high cognitive and physical functional capacity, and active engagement with life” [9] (p. 433). However, the notion of successful ageing has some significant limitations. Firstly, successful ageing portrays an idealistic and unrealistic view of older age and what can be achieved by many older adults [10]. Further, it ignores many of the socio-economic and other intersecting factors that may contribute to someone’s ability to ‘age successfully’. Additionally, the idea of successful ageing inadvertently suggests that older adults who cannot reach this ideal have ‘unsuccessfully aged’ [11]. 

In light of these limitations, as well as the broader scope permitted by the notion of positive ageing, this paper takes positive ageing as its focus and explores how the role of religion, spirituality and/or belief can be incorporated into it. In addition to the definition from Stock et al. [7], positive ageing is considered here to be a multidimensional approach to the everyday life of older adults, which is unique to the individual rather than an optimal state of being to be achieved. As such, it may encompass many more factors that are considered important to the individual and these may play out in different ways for different people. 

Religion is often seen as a public and outward form of expression whereas spirituality is generally seen as something inward and personal. Koenig [12] argues that religion is often related to the idea of the ‘transcendent’, with certain beliefs, practices and behavioural rules associated with it; it is organised and often community-based, but can also be practiced privately. The modern notion and understanding of spirituality, however, includes not only people who follow a particular religion, but also those who do not. Spirituality is understood as ideas surrounding “a sense of connectedness, purpose, meaning and ‘transcendence of self’’’ [13] (p. 106), and is highly personal [14]. Lastly, the notion of belief is widely understood as a “common and an essential part of ordinary living” [15] (p. 1). Coleman argues that beliefs “determine what we value as goals and objectives in life” [15] (p. 1). Belief may involve both religiosity and spirituality, but it can also be linked to cultural values (see Day [16] for an in-depth discussion of ‘belief’). However, in this paper, the use of ‘belief’ is limited to discussions of religious and spiritual values.

Religion, spirituality and/or belief have been seen as the lenses through which some people interpret, understand, evaluate and respond to their experiences in the world and give people a sense of meaning and purpose in life [17]. As such, Schlehofer, Omoto and Adelman emphasise the importance of people’s lay definitions and understandings of what religion, spirituality and/or belief mean to them and how they relate to their everyday realities [18]. Therefore, all three terms are used broadly in this paper and are not to be conflated or taken as synonymous with each other. Rather the terms are used in order to be as inclusive as possible of the wide variety of lay definitions and understandings. As mentioned, the focus here is on conceptual understandings within Western society, but it is important to note that different understandings of religion, spirituality and/or belief exist elsewhere and are not all based on Judeo-Christian conceptions (e.g., see Ahmad and Khan [19] on Islamic spirituality).

### 1.2. Context of Complexity and Change for Religion, Spirituality and/or Belief

The conceptual understandings of religion, spirituality and/or belief emphasise their complexity and the diversity of possibilities of how they may play out in people’s everyday lives. Something to keep in mind is the significant complexity and changes in religious and spiritual affiliation and practice, a shift from the public to the private sphere (especially within European countries) [20]. In England and Wales, the 2011 Census [21] highlights some of the key changes in relation to religion. Christianity remained the largest religion with 59.3 per cent of the population identifying as Christian; however, this figure had decreased from the 2001 census where 71.1 per cent of the population identified as Christian. The second largest group in 2011 were Muslims, making up 4.8 per cent of the population. In addition to those affiliated with a particular religion, 25.1 per cent reported no religion and 39,000 people identified with spiritualist groups. It is likely that these trends have progressed even further in recent years and although these statistics do not specifically refer to older adults, research shows that older adults tend to be more religious than younger generations and some claim that religiosity can increase with age [22]. Moreover, behind these statistics is a wide array of ways in which people engage with religion, spirituality and/or belief across time and circumstance. For example, many people may identify with a religion but do not actively engage in religious practices, while others may not identify with a religion but still draw on religious support in times of need. 

The shift of religion, spirituality and/or belief from the public to the private sphere is evident in almost all Western European countries. This significant change in the religious landscape is highlighted by Taylor, with regards to political structures: “Churches are now separate from political structures (with a couple of exceptions …). Religion or its absence is largely a private matter. The political society is seen as that of believers (of all stripes) and non-believers alike” [23] (p. 1).

A lot of the past research has been conducted in the United States of America (USA) [24,25], whereas the current study is based in the United Kingdom. Berger, Davie and Fokas illustrate how “[t]he American picture differs sharply from the European one. Both behavioural and opinion indicators are much more robustly religious” [26] (p. 12)*.* Taylor provides an in-depth discussion around some of the reasons for these differences [23] (pp. 526–535). However, despite shifts in the nature of religion, spirituality and/or belief in everyday life in the UK, these influences inevitably still play a significant role in the private lives of many.

Research has shown that religion, spirituality and/or belief are intertwined with health, well-being and quality of life, particularly with regards to how people deal with ill-health and other challenges they might face. Indeed, Koenig notes that it is only in fairly recent times that religion has become disconnected with healthcare, despite the influence it can have on medical decisions, the kind of support structures available, and the capacity to cope with ill-health [27]. Occupational therapy, for instance, initially encompassed religion and spirituality into a holistic approach to the body, mind and spirit of the individual and allowed a more personal relationship between therapist and patient [28]. However when more scientific models of medicine became dominant, attention to religious and spiritual needs declined. Currently in the UK, incorporation of religious and spiritual needs in the National Health Service (NHS) is sporadic, although it is present as part of end-of-life care and sometimes within mental health care [29,30]. Meanwhile in Scotland, NHS staff are required to respect “the dignity, humanity, individuality and diversity of the people whose cultures, faiths and beliefs coexist in Scottish society” [31] (p. 5) and recognise “faith, hope and compassion in the healing process” [31] (p. 7). The relationship between religion, spirituality and/or belief and healthcare may have pertinent implications for older adults in terms of improving the patient experience and facilitating a faster recovery both physically and mentally.

The complexity and change associated with religion, spirituality and/or belief has particular consequences for older adults, who are likely to have grown up with strong religious values that dominated the public sphere, but have seen a decline in religious affiliation in the generations following them, resulting in changes in society and the shift of religion into the private sphere. Many older adults have held onto their faith or may have even grown more religious as they age [22,32]. For example, Balboni et al. reported that as people’s physical health decreases and they become more familiar with end of life and mortality, religiosity and spirituality increases [5]. Religion, spirituality and/or belief could therefore play an important role in their everyday lives and moreover, it may be supportive in the challenges associated with ageing [33].

### 1.3. Religion, Spirituality and/or Belief for Health, Well-Being and Positive Ageing

Though there is little research specifically linking religion, spirituality and/or belief to positive ageing, some literature does attend to spirituality and successful ageing, as well as the health and well-being of older adults more generally. Crowther et al. discuss the notion of ‘positive spirituality’ by building upon Rowe and Kahn’s model of successful ageing [9], which they argue ignores the important role spirituality plays for older adults in the ageing process [34]. They state that:

*Positive spirituality involves a developing and internalised personal relation with the sacred or transcendent that is not bound by race, ethnicity, economics, or class and promotes the wellness and welfare of self and others. Positive spirituality uses aspects of both religion and spirituality*.

[34] (p. 614)

They go on to suggest that the notion of positive spirituality can decrease some of the feelings of helplessness and loss of control that people experience with illness, as well as reduce stress and bring about increased feelings of purpose in life. They argue that spiritual activities (e.g., prayer) can reduce feelings of isolation and the community aspect surrounding spirituality can have positive outcomes for older adults. Similarly, Sadler and Biggs argue that understanding existential and spiritual needs of older adults would increase the understanding of so-called ‘success’ in later life [35]. They suggest that acknowledging spirituality is important because it may influence well-being in later life and allow older adults to adjust accordingly with some aspects of growing older. Moreover, having a positive perspective, being able to cope, having active independence, meaningful relationships, freedom, having a relationship with God and a sense of spirituality were some of the aspects cited by older adults as contributing to ‘successful ageing’ [36]. Though this literature uses the term successful ageing, the ideas discussed are pertinent to the more inclusive concept of positive ageing and demonstrate the possibilities for religion, spirituality and/or belief to promote positive ageing.

The following literature shows the contribution religion, spirituality and/or belief has to positive ageing in helping older adults to cope with adversity, providing social support and opportunities for participation in society, and enhancing well-being. Although some of the literature presented here does not focus exclusively on older adults, the health issues they address are widely associated with older age. Religion, spirituality and/or belief were found to be key components for individuals when coping with the diagnosis and management of a disease in general [37,38], cancer and chronic illness [39], arthritis and cardiovascular disease [40], and also dementia [4]. Further, Manning’s research shows how older women used their sense of spirituality as a tool of resilience which helped them to cope with adverse life challenges and to remain optimistic, thus contributing towards a sense of well-being [2]. Religion, spirituality and/or belief were also found to provide social support, connectedness to others and a sense of belonging to a community for older adults in general [27], older adults with physical health conditions [5,38,41], and older adults with mental health conditions including anxiety and depression [42] and dementia [4]. Moreover, George et al. found how religion, spirituality and/or belief can provide supportive communities and reduce feelings of loneliness and social isolation experienced by many older adults, as well as giving them a sense of meaning in their lives [43]. The idea of religion, spirituality and/or belief offering meaning and purpose in life has also been found for older adults with health conditions [37,40], dementia [4], and when preparing for end of life [41]. Harvey found that spirituality offered a sense of faith, meaning, understanding, purpose, inner peace and well-being, and allowed older adults to feel a connection with others and with a higher being [40]. Finally, Manuti argues that religion “could be considered as a meaningful life domain which gives further sense to ageing experience” (p. 11) and suggests that religion and spirituality can act as a major cultural resource for the identity of many older adults [44].

Despite these clear links and the importance of religion and spirituality to many, Nelson-Becker et al. found that it is too often absent from care and, in particular, end-of-life care [13], as well as mental health care [30]. Similarly, it is also missing from the discourse on positive ageing. Though many of the factors covered above (e.g., increased well-being and participation in society) are discussed in greater depth in the edited volume on positive ageing from the Centre for Positive Ageing [45], there is no mention of religion, spirituality and/or belief.

### 1.4. Gap in the Literature and Scope of the Study

As the above research has shown, the role of religion, spirituality and/or belief can have numerous positive outcomes for older adults including enhanced health and well-being, greater capacity to cope, social support, and opportunities to participate in society. We argue that these are issues which could enhance the understanding of the role religion, spirituality and/or belief play in debates around positive ageing. However, there are a number of limitations in the existing literature that indicate a need for further and specific research in this area. 

Firstly, a significant amount of the literature is based on quantitative research. Using qualitative methodology allows for deeper understandings of such abstract concepts of religion, spirituality, belief and positive ageing to emerge. Secondly, much of the literature focuses on Christianity [14] in the USA [24]. The religious and spiritual environment in the UK is hugely diverse and very different from that of the USA, as is the healthcare approach to ageing. Thirdly, although some studies have looked at religion, spirituality and/or belief in relation to successful ageing, the link between religion, spirituality and/or belief and positive ageing as a more inclusive concept has not yet been developed in the literature. This is important as the possibilities for support through religion, spirituality and/or belief may have compelling implications for positive ageing in our current situation of an ageing population during a time of austerity in the healthcare sector. In light of these limitations, this paper aims to qualitatively explore the role of religion, spirituality and/or belief in the everyday lives of older adults in the UK to understand how it may be related to positive ageing. The methodology and findings from a qualitative exploratory study will be presented, followed by a discussion including initial suggestions for practice and further research.

## 2. Materials and Methods

### 2.1. Sample

A gatekeeper provided initial contact with a forum for older adults based in West London. The forum is a non-religious association that aims to address issues affecting older adults in the borough, for example social isolation, local health services and transport. The opportunity to take part in one of two focus groups exploring the role that religion, spirituality and/or belief played in their everyday lives in relation to ageing was presented to the members of the forum, and they were able to ask the researchers any questions before deciding whether to participate. All members who were interested were invited to take part, regardless of their religion, spirituality and/or belief, resulting in a total of 14 participants, three male and 11 female. The age of participants ranged from 63 to 92 years of age (although one participant did not declare their age). Three participants described themselves as having no religious or spiritual belief, two participants were Muslim, one participant was Jewish, five participants described themselves as Christian (including three Roman Catholic, one Church of England and one Adventist), and the remaining three participants described their religious or spiritual affiliation as a mixture of things including ‘Catholic/Buddhist/nature’, ‘Christian/Buddhist’, and one stating ‘I only see God in all things good, amazing and beautiful’.

### 2.2. Data Collection

Focus group discussions were chosen because they allow for discussion and debate to take place between a number of participants. As Hennink states, as a “discussion proceeds participants begin to ask questions or clarifications of others in the group, which may trigger them to raise additional issues or share similar experiences, thus increasing the clarity, depth, and detail of the discussion” [46] (p. 3). The first focus group comprised eight participants (participants 1–8) and lasted 1:08:44 and the second focus group consisted of six participants (participants 9–14) and lasted 1:17:47. Focus groups were facilitated by the first author and notes were taken by the second author. Participants were asked a series of questions including: How would you describe what spirituality, religiosity or a sense of belief mean to you?; How would you describe the role your religious and/or spiritual belief has on your day-to-day life?; Have your beliefs changed over time?; Have your beliefs, or your sense of religiosity/spirituality ever helped with any aspects or challenges of growing older?; Have your beliefs ever been a barrier?; When it comes to your health and well-being—how important do you think it is to take your religious and spiritual beliefs into account?; Have your spiritual or religious needs ever been addressed by health and social care professionals, or should they be? Questions were followed by prompts if needed and additional questions were asked depending on the direction of conversation.

### 2.3. Ethical Considerations

Ethical approval for this study was approved by the Department Research Ethics Panel (DREP) in the Faculty of Health, Social Care and Education at Anglia Ruskin University (FHSCE-DREP-16-092). Participant information sheets and consent forms were given to those taking part and discussed before informed consent was obtained. Given the sensitive nature of discussing personal experiences of religion, spirituality and/or belief, as well as life challenges associated with older age, ground rules were established for the focus group discussions. These included listening to each other, respecting each other’s points of view, and the option to stop and/or leave at any time without giving a reason. Participants received a £10 high street shopping voucher as a thank you gift for taking part. All identifiable information has been anonymised to protect those involved in the study. 

### 2.4. Analysis

With consent from participants, the focus groups were audio-recorded and subsequently transcribed verbatim. A thematic analysis informed by Braun and Clarke [47] was conducted on the data to identify key themes relating to the role of religion, spirituality and/or belief in positive ageing for older adults. Thematic analysis was selected as an appropriate method as it allows extensive textual data to be summarised and organised without compromising on rich description, and therefore keeping the experiences, lived reality and meaning of participants evident in the research. Braun and Clarke’s six-stage process includes:

1. Familiarisation with the data
2. Coding
3. Searching for themes
4. Reviewing themes
5. Defining and naming themes
6. Writing up

Through reading and re-reading through the transcripts, as well as listening to the original recordings, the first author familiarised themselves with the data, then coded and identified initial themes, which were then checked and refined collaboratively with the second author. A total of five themes were identified and the findings are presented in the form of a summary of these themes evidenced with illustrative quotes.

## 3. Results

The thematic analysis identified five common themes relating to religion, spirituality and/or belief and positive ageing across the data: changing religious landscapes in a modern world; the personal and interpersonal nature of religion, spirituality and/or belief; a source of strength, comfort and hope; sense of community and belonging; and the need for a holistic approach.

### 3.1. Theme One—Changing Religious Landscapes in a Modern World

The first theme focused on the changing nature of the religious landscape in the modern world. A number of participants expressed how their experience and relationship with religion has changed significantly since they were younger and that some of the behaviours and ways of living associated with religion were changing and in some cases declining, with other activities becoming more important in everyday life, such as shopping on Sundays instead of going to church. This was articulated by participants in the following quotes:

… in the wartime at nine o’clock we used to have the news at nine o’clock and when Big Ben was striking, everybody prayed. I mean that was the kind of ethos. We had days of prayer, do you remember? I’m sure, everyone, (laughter) through the wars, days of prayer, which were held, I presume in all kinds of denominations in Christianity, perhaps Roman Catholic churches, I don’t know. But you wouldn’t hear of anything like that happening now would you?

(Participant 5)

And I think that’s one of the downsides of religion these days. And it comes into the fact that instead of going to church on Sunday, I’m only saying it, I’m not judgemental or anything, that the shopping has taken over (laughter) a little bit.

(Participant 8)

Another aspect of this was the inability to relate to the manifestations of religion that were apparent in some parts of the world today. For example, one Muslim participant discussed her experience of growing up in Iraq where the religious and cultural norms that are prominent today were not a part of the multi-religious and in many parts secular environment in which she grew up:

One common denominator, I think, it was only the elderly people that went to a Mosque or even prayed, I mean quite a few didn’t even go to a Mosque … So it’s a secular environment, not very religious and religion wasn’t playing a very important dominant role … I’m a product of that, I think. I don’t wear the veil. It’s quite alien, you know, this is very new.

(Participant 9)

There was discussion about the perceived decline in pro-social behaviours, for example manners and common courtesy, which the participants associated with religious values as well as the diminishing importance of the family in modern society as a consequence of the changing religious landscape.

… It’s the family ties that no longer exist and a lot of the breeding and civility starts at home with your parents, with your … Do you know, even the little things, okay time doesn’t permit it but getting together with the family, having a meal, I mean this is something, okay life has changed, people have different obligations, different places to be.

(Participant 10)

Another participant talked about a religious tradition to look after the elderly, and how they had cared for elderly family members towards the end of their life, but this tradition was ‘gradually eroding’. In terms of healthcare, one participant who was a retired nurse explained how every aspect of a person’s life used to be taken into account when they were admitted as a patient, including their social life, religion and family situation; something that she claimed has changed in today’s world.

### 3.2. Theme Two—Personal and Interparsonal Nature

The second theme emphasised the idea of the personal and interpersonal nature of religion, spirituality and/or belief. Numerous participants explained how religion, spirituality and/or belief is something private and personal, and that people can have an internal, direct relationship with ‘God’ (or another higher being or spirituality) regardless of their religion:

I don’t need a middleman to be spiritual. I think it’s just a direct relationship and maybe this is why I don’t go, I don’t feel the need to go to a Mosque or to see a Cleric or anything of that kind. So for me I think a family member would be just perfect, I don’t need any preaching from anybody and I’m not preaching anybody. I’d prefer it without.

(Participant 9)

Moreover, many participants suggested that religion, spirituality and/or belief was about your intrinsic values and how you treat other people on an interpersonal level. For many, being religious was not evident through going to a place of worship every week, wearing a veil or praying numerous times a day; rather it was demonstrated through people’s actions, through being considerate, kind and loving. In this way, they saw religion as a guide to live life a certain way:

About going to church, that doesn’t mean religion—some people think that that is religion, but to me—I mean, generally, it is not the religion … No. You have to be a good—spiritually Christian or Muslim or Jewish, you have to be that. You [show yourself] from your lifestyle, not what others do.

(Participant 6)

Then we were talking about treating each other with respect and I thought the old thing, the old golden rule. If you won’t live by the old golden rule, that covers every religion, that, really you know—do unto others as you’d have them do unto you.

(Participant 12)

On the other hand, for one participant, a Catholic nun, the outward expression and adherence of her faith was fundamental and praying throughout the day as well as teaching others about the Catholic faith was of central importance in her life. Overall, the notion of treating others kindly and considerately for the majority of participants was positive as it brought about their own sense of well-being as well as potential reciprocal interpersonal relationships with others. 

### 3.3. Theme Three—Source of Strength, Comfort and Hope

The third theme identified was religion, spirituality and/or belief as a source of strength, comfort and hope. When facing a difficult or challenging time in their life, some participants drew on their religion, spirituality and/or belief to help them through and give them the hope that everything would be okay.

I’m 76, I used to be incredibly healthy and energetic and vigorous and the older I get the more limited I am physically. I’m more mature and developed spiritually and mentally but not physically and I forget a lot. I find it … The only way I can cope, the only way I can cope is through prayer and the love of God, that’s the only way.

(Participant 13)

It could also bring a sense of comfort and solidarity with others, as expressed by one participant who described how in times of desperation and need she would say certain phrases like “God be with you” in Arabic or Farsi. Another participant felt that religion, spirituality and/or belief could provide hope in challenging times:

Yes, for me, in terms of catastrophes like for example in Iraq the various wars that I’ve been through for the Iran-Iraq War, the Gulf War, then second, the last one and the sanctions, I think it [religion, spirituality and/or belief] does play an important role. You need, when you’re sinking you need something to clutch on to, some hope, some miracle, you know, anything that just gives you that light at the end of the tunnel kind of thing.

(Participant 9)

Furthermore, in discussing some challenging legal issues she had faced, one participant explained how solidarity and support transcended the barriers between different religions:

I just went through different religious kind of centres and some Indians, some Protestants and all the people, they all said they were going to pray for me. And I liked that. (Laughter). And so, after I won my case, I just wanted to thank them … So I didn’t care which religion, but somehow all these places all said they going to pray for me (laughter).

(Participant 7)

Religion, spirituality and/or belief in older age was also linked to comfort, hope and peace of mind in relation to ill-health in older age. Although most participants believed that it could not cure physical illness, they talked about how religion, spirituality and/or belief could give people something to hold on to, calming anxiety and allowing for tranquillity:

So I think that perhaps the spirituality can affect that, can give that calmness and peace of mind. I think from that point of view that probably is quite … I don’t think it could affect your aches and pains and so on … No I think from that point of view, peace of mind, tranquillity really, yes, I suspect that spirituality is helpful in that way, yes.

(Participant 11)

In this way, participants suggested that religion, spirituality and/or belief could be a positive influence and contribute towards a quicker recovery from ill-health in older age by providing inner strength, comfort and hope, which could act as key components of positive ageing. 

### 3.4. Theme Four—Sense of Community and Belonging

The fourth theme was the sense of community and belonging associated with religion, spirituality and/or belief. This was discussed in terms of being part of a religious community and participating together in religious practices and traditions, such as going to church and praying together. However, beyond this, religion, spirituality and/or belief also served as a canvas for social activity which can lead to companionship, for example sharing a meal together.

I think, the fact that churches do form a community in London and everywhere, really. That you do meet other people, you get to know other people, you make friends that when I first came to London I went to a church near here and there was a group of young people and we’d all arrived in London, didn’t know people there so we all became very friendly. It provided a focus as well as the actual religious thing, as the forum this afternoon provide a focus for people. Removes the sense of isolation perhaps but I think it is there’s a sense, perhaps, of our inadequacy, a comfort.

(Participant 11)

Participants linked this sense of companionship and community to addressing isolation and loneliness, which is a particular problem for older adults. This was also prevalent through less obvious connections to others of the same faith, for example one participant stated:

I don’t actually go to church, but every morning I try to listen to the daily service, which I feel all over the country people are listening, I don’t feel alone at all [with] the Sunday morning service.

(Participant 5)

However, one participant cautioned of the negative consequences of religious and/or community groups particularly for older adults:

Or this gentleman said he liked his group and blah, blah and the familiarity of safety in old age and … That’s a very dangerous place for me to be in because once the group breaks up whatever the reason—dying or there’s a bomb falling on it and the society disseminates, I’m abandoned by God.

(Participant 14)

Nonetheless, on the whole, the sense of community and belonging that can arise from religion, spirituality and/or belief was positive and welcomed and for some, coming together with other people was considered as religious in itself. This further highlights the role religion, spirituality and/or belief can play in positive ageing, allowing for participation in society and enabling increased well-being. 

### 3.5. Theme Five—The Need for a Holistic and Individual Approach

The last theme highlighted that religion, spirituality and/or belief should be a choice and never imposed. A number of participants talked about their childhood and upbringing and how religion was often imposed upon them, for example through the education system or through their parents taking them to church. In some cases, this led them to turn away from the organised religion they were brought up with, although many retained a sense of spirituality and/or belief.

My upbringing is rather like yours, a boarding school where there was too much religion and I think I took the easy way out of getting confirmed, because I didn’t want to confront my head mistress. So that lasted about a couple of years, you know, going to communion and things.

(Participant 1)

Discussions around choice and imposition centred on the way in which religion, spirituality and/or belief is involved in healthcare, particularly in end-of-life care. This caused a divide in the participants, where some stated firmly that they would not want their religious or spiritual needs addressed in a healthcare setting while some stated firmly that they would, and others had no preference. This highlighted the need for individual choice in whether religious or spiritual needs are addressed during end-of-life care, as one participant explained, she hoped that her views and wishes would be respected when the situation arose.

Some participants struggled to understand how healthcare professionals could stay neutral in certain situations, for example, if someone’s religious or spiritual needs contradicted with the healthcare professionals’ duty of care. There was also a concern for some that healthcare professionals would not be neutral and that healthcare staff would be taking on a role they were not equipped for, and these participants would prefer that their religious and/or spiritual beliefs stayed separate from their healthcare. 

A number of participants shared their own experiences of healthcare and how generally religion, spirituality and/or belief was not mentioned. All emphasised the importance of asking older adults what they want and one participant in particular stated it ‘would have been nice’ if her religious and spiritual needs were taken into account:

Now the right thing for, I think for old people is to ask the old people themselves what do they want? They consider that because you’re old, you’ve got dementia, you don’t know what you want it’s not, your thinking is not good enough.

(Participant 14)

Essentially, the participants indicated their desire for a more personal and holistic approach to their health and well-being, where healthcare professionals understood more about the life of the older adult, which could include religion, spirituality and/or belief if it is considered important for the individual:

But I think there should be some [consideration of religion, spirituality and/or belief]. I mean when you go to see a homeopathic practitioner, they spend an hour with you and they talk to you about all sides of your life, your interests, what you’re doing and it’s wonderful … You feel that somebody has not just sat there for 10 minutes writing a prescription (laughter). They give you this lovely feeling that you are the point of interest and that you have such meaning and that your whole life is playing on your health, affecting your life, your lifestyle, your beliefs, everything.

(Participant 5)

Having the choice as to whether religion, spirituality and/or belief is addressed in healthcare, and choice in everyday life as older adults more generally, was vital for participants, and this also adds towards ideas of positive ageing as it helps promote independence and dignity for older adults.

## 4. Discussion

The themes that emerged from the data show the multifaceted ways in which religion, spirituality and/or belief manifest themselves in the everyday lives of older adults and influence their experiences of ageing. The first theme of changing religious landscapes in a modern world highlights those changes that were considered most important by the participants, such as the perceived breakdown of traditional family values that were shaped by religion. These changes go beyond the statistics on the decline in religious affiliation in England and Wales [21] to discuss some of the consequences for positive ageing, such as the breakdown of family ties and care for the elderly increasingly no longer taking place within the family. Participants also noticed how times have changed in terms of incorporating religion into healthcare, which like Koenig [27], they claimed used to happen in the past but is now rare. This points towards the poignancy of this research in terms of how these changes influence the positive ageing of older adults and the need to go beyond specific health issues to incorporate factors such as religion, spirituality and/or belief. The second theme indicates the intrinsic personal and interpersonal nature of religion, spirituality and/or belief for older adults and the way in which it is bound up in pro-social behaviours, which was considered by most participants as more important than any outward profession of faith. Interestingly, this theme did not cover the changes related to the state [20] or political structures [23] considered in the literature, but focused more on the social changes in behaviour as a result of the changing religious landscape and individual experiences of religion, spirituality and/or belief as a private and personal matter. As such, themes one and two reflect on the contextual factors of complexity and change for religion, spirituality and/or belief from the perspective of older adults and point toward the factors that are most important for them, for example, family life and interpersonal as well as intergenerational relationships. 

Theme three demonstrates how religion, spirituality and/or belief can provide a source of strength, comfort and hope, especially when experiencing health issues and other challenges associated with ageing. These factors are understood as contributors to positive ageing and therefore demonstrate a role for religion, spirituality and/or belief in positive ageing. For example, participants talked about how it offers a sense of calm and peace of mind during illness and one participant stated that prayer was the only way she could cope. Indeed, these intrinsic factors of strength, comfort and hope could be seen as different mechanisms by which religion, spirituality and/or belief help older adults to cope more positively with the challenges they face. This is in line with the literature that identifies a role for religion, spirituality and/or belief as a source of resilience and coping [2,36]. Park states that religious coping “involves activities such as trusting in God’s love … seeking spiritual support, and seeking support from members of one’s religious group” [17] (p. 323). The idea of religious coping in both the data and the literature has important implications in terms of strengthening approaches for positive ageing.

Similarly, theme four supported the literature that indicates how religion, spirituality and/or belief can provide social support through community and belonging for older adults. For example, participants discussed how belonging to a religious or spiritual community helped them to feel less alone and isolated, as found by Crowther et al. [34] and George et al. [43], by taking part in religious and social activities, as well as simply knowing that others out there are part of that community. This theme can also be considered in relation to elements from theme one around the diminishing role of the traditional family and what this means for the social support networks for older adults, and how the changing modern world was seen as a threat to the sense of community. Nonetheless, themes three and four echo the literature in recognising the potential for religion, spirituality and/or belief to promote positive ageing in the face of challenges through intrinsic coping mechanisms of strength, comfort and hope as well as extrinsic support from belonging to and participating in a community. 

The final theme highlighted the importance of choice and holistic approaches for older adults in terms of how religion, spirituality and/or belief are incorporated into their lives and into approaches to positive ageing. Given the differences of opinions on whether religious and spiritual needs should be addressed in healthcare, it is clear that choice is paramount. Although there are numerous recommendations on how religion and spirituality should be addressed within the NHS, McManus argues that, in reality, these issues remain misunderstood and are not always integrated efficiently into practice [48]. Koenig [27] and Wilkinson and Coleman [6] note that healthcare professionals should remain neutral and respectful when addressing issues of religion and spirituality. As mentioned above, the neutrality of healthcare professionals was something that concerned participants, thus for many, having their religious or spiritual needs addressed in this setting was not appropriate. Therefore, perhaps a general, but consistent approach within healthcare to respect and incorporate any religious or spiritual needs for those who want it should be practiced to enable people to get the right care and help they need in an individualised and person-centred way. Having this choice may be a crucial component for positive ageing for older adults; participants stressed that health professionals should ask older adults about what they want more generally when their health is at stake and it should not be assumed that older adults are incapable of making their own decisions. In this way, participants advocated for a more holistic approach to healthcare in older age that treats older adults as individuals with many aspects of their life shaping their health, which may include religion, spirituality and/or belief. This allows for not only the inclusion of religion, spirituality and/or belief in approaches to positive ageing, but also a variety of other factors that influence the ageing process but may have been overlooked. The importance of having a holistic and individual outlook for positive ageing is evident across a number of aspects related to the ageing process. For example, Stewart et al. discuss the need for an individualised approach for older adults in terms of housing issues [49]; Bacon and MacKinnon stressed the importance of having an individualised/personalised approach in terms of digital technology for older adults [50]; and Bellamy highlighted the need for individual choice in terms of end-of-life care [51]. Thus, this personalised approach and recognition of the diverse needs of older adults holds importance not only in healthcare, but in all aspects of life allowing older adults to age more positively. 

There are a number of limitations to the research that need to be taken into account, but also identify areas for future research. Given that the research was an exploratory study, the size of the participant group, gender imbalance and the fact that they were all recruited from a forum based in an affluent borough of West London undermines the representativeness of the views expressed. As such, research with more diverse groups of older adults from different parts of the country would be useful in ascertaining the role religion, spirituality and/or belief has in their lives and experiences of ageing. In addition, some participants found it difficult to imagine the ways in which religion, spirituality and/or belief could be addressed in healthcare, beyond its incorporation into end-of-life care. Future research could delve deeper into these possibilities by searching for examples and developing new approaches to healthcare and positive ageing that consider religion, spirituality and/or belief. Furthermore, it is important to note that religion and spirituality do not always have a positive effect in people’s lives. Though not prominent in the current study, other research has highlighted that in ill-health, people may suffer from feelings of abandonment or punishment by God or a higher being [43,52] or may abdicate responsibility which could further jeopardise health [14]. These issues emphasise the need for caution as well as choice when utilising religion, spirituality and/or belief to promote positive ageing and the risk of it hindering positive ageing or indeed being harmful should be carefully managed. 

Despite these limitations, the research has valuable implications for practice when working with older adults in the UK. In healthcare, older adults should be treated as a whole person with a wide range of factors that influence their health. Given the personal nature of religion, spirituality and/or belief and the importance of choice, older adults should be asked about what they feel is important, which may be social, economic, cultural or religious and spiritual factors, amongst others. This should be consistent across all services and visits, genuine rather than a tick box exercise, and flexible to allow for older adults to change their minds. In terms of positive ageing, approaches should include a consideration of how religion, spirituality and/or belief influence the everyday lives of older adults. The intrinsic and extrinsic support available through religion, spirituality and/or belief is not only a valuable resource in itself for positive ageing, but also provides a starting point for thinking more creatively about different forms of support for older adults through the ageing process. For example, the Local Government Association has recently explored how working with faith groups can help promote health and well-being, and highlights how increased pressure on the NHS could mean that faith groups become more centrally important when it comes to health and well-being promotion [53]. These findings support this idea and suggest that it would be particularly fruitful in promoting positive ageing for older adults. 

In line with the definition of positive ageing provided by Stock et al. [7], the findings from this study illustrate the important role religion, spirituality and/or belief has in promoting positive ageing for older adults. As the definition states, positive ageing “denotes the aspirations of individuals and communities to plan for, approach and live life’s changes and challenges as they age and approach the end of their lives in a productive, active and fulfilling manner” [7] (p. 5). This was highlighted in a number of ways by participants, for example religion, spirituality and/or belief provided a basis for how participants approached and lived their lives, as seen with the discussion around treating people in a considerate, kind and loving way was vitally important. This had subsequent consequences for how they felt about their own lives, as one participant articulated ‘do unto others as you’d have them do unto you’. Moreover, the role of religion, spirituality and/or belief provided a source of strength, comfort and hope which allowed participants to deal with life’s changes and challenges as they aged.

The definition of positive ageing also stresses how positive ageing embraces things which promote “a person’s sense of independence, dignity, wellbeing, good health and enable their participation in society” [7] (p. 5). These findings show how religion, spirituality and/or belief often enhanced participants’ sense of well-being, for example, through lessening anxiety, bringing about peace of mind and helping participants cope with difficult circumstances in their lives. Furthermore, in line with the definition of positive ageing, religion, spirituality and/or belief was found to enable participation in society. This is evident in theme four as participants described how religion, spirituality and/or belief could bring about a sense of community and belonging and could act as a canvas for social activity, which in turn made some participants feel less lonely and isolated. However, as shown in the findings older adults have complex and changing relationships with religion, spirituality and/or belief and it is not a panacea, therefore religion, spirituality and/or belief should be seen as one form of support within a plethora of others (both formal and informal) for a comprehensive approach to positive ageing.

## 5. Conclusions

This research aimed to stimulate the discussion around the potential for religion, spirituality and/or belief to promote positive ageing in the UK. By drawing on the thoughts and experiences of older adults, we have shed light on how religion, spirituality and/or belief can have a significant influence on the everyday life of older adults and how it can be understood within the wider concept of positive ageing. The narratives discussed highlight the continuing importance of religion, spirituality and/or belief despite the changing nature of religion; the personal and interpersonal nature of religion, spirituality and/or belief for older adults; the positive influence it has as a source of strength, comfort and hope in times of need; the sense of community and belonging it can provide in a time of heightened risk of loneliness and social isolation; and the possibilities for including religion, spirituality and/or belief into a more holistic approach towards the health, well-being and lives of older adults. In conclusion, there are broad and valuable implications for the role of religion, spirituality and/or belief in promoting positive ageing. Research and practice around positive ageing should include a consideration of religion, spirituality and/or belief in order to find new ways to promote positive ageing. Moreover, the discourse on positive ageing should incorporate a consideration of religion, spirituality, and/or belief in order to fully understand the experience of older adults and promote their health, well-being and quality of life. Overall, acknowledging and drawing on the various roles that religion, spirituality, and/or belief play in the lives of older adults can help them in turn to age positively and live their lives in a fulfilling way. 
